# Effects of Isorhamnetin in Human Amniotic Epithelial Stem Cells *in vitro* and Its Cardioprotective Effects *in vivo*

**DOI:** 10.3389/fcell.2020.578197

**Published:** 2020-09-29

**Authors:** Kazuhiro Aonuma, Farhana Ferdousi, DongZhu Xu, Kenichi Tominaga, Hiroko Isoda

**Affiliations:** ^1^School of Integrative and Global Majors (SIGMA), University of Tsukuba, Tsukuba, Japan; ^2^AIST-University of Tsukuba Open Innovation Laboratory for Food and Medicinal Resource Engineering (FoodMed-OIL), AIST, University of Tsukuba, Tsukuba, Japan; ^3^Alliance for Research on the Mediterranean and North Africa (ARENA), University of Tsukuba, Tsukuba, Japan; ^4^Cardiovascular Division, Institute of Clinical Medicine, Faculty of Medicine, University of Tsukuba, Tsukuba, Japan; ^5^Faculty of Life and Environmental Sciences, University of Tsukuba, Tsukuba, Japan

**Keywords:** human amniotic epithelial stem cells, drug screening, isorhamnetin, cardiac fibrosis, angiotensin II, translational medicine

## Abstract

Cardiac hypertrophy and fibrosis are major pathophysiologic disorders that lead to serious cardiovascular diseases (CVDs), such as heart failure and arrhythmia. It is well known that transforming growth factor β (TGFβ) signaling pathways play a major role in the proliferation of cardiac hypertrophy and fibrosis, which is mainly stimulated by angiotensin II (AgII). This study aimed to investigate the cardioprotective potential of isorhamnetin (ISO) in human amniotic epithelial stem cells (hAESCs) through global gene expression analysis and to confirm its beneficial effects on cardiac hypertrophy and fibrosis in the AgII-induced *in vivo* model. *In vitro*, biological processes including TGFβ, collagen-related functions, and inflammatory processes were significantly suppressed in ISO pretreated hAESCs. *In vivo*, continuous AgII infusion using an osmotic pump induced significant pathological fibrosis and myocardial hypertrophy, which were remarkably suppressed by ISO pretreatment. ISO was found to reverse the enhanced TGFβ and Collagen type I alpha 1 mRNA expression induced by AgII exposure, which causes cardiovascular remodeling in ventricular tissue. These findings indicate that ISO could be a potential agent against cardiac hypertrophy and fibrosis.

## Introduction

Pathological cardiac fibrosis is a fundamental process in the excessive accumulation of extracellular matrix (ECM) such as collagen (Travers et al., 2016), which plays a key role in the disruption of the myocardial architecture, myocardial disarray, and electrical and mechanical cardiomyocyte dysfunction (Izawa et al., 2005). In addition, the loss of normal ECM structure impairs the integrity of cell-to-cell contraction, further isolates myocardial fibers, impairs oxygen supply, and causes atrophy and necrosis (Kai et al., 2006). Thus, cardiac fibrosis leads to various heart diseases, including cardiac hypertrophy, arrhythmia, and heart failure (Weber et al., 2008; Yue et al., 2011) and is considered an independent risk factor for cardiac morbidity and mortality (Bang et al., 2017; Jin et al., 2018). Controlling hypertrophic remodeling and fibrotic changes may therefore offer a promising therapeutic strategy for reducing the cardiovascular disease (CVD) burden.

In recent years, increasing numbers of small molecules derived from or based on bioactive compounds of medicinal plants have been synthesized and screened for their potential therapeutic and preventive effects in CVD. However, the discovery of drugs is greatly hampered by the gap between the validation of the compound and its successful clinical application (Pott et al., 2020). One of the primary reasons for this translational inefficiency is the unpredictability of the currently used *in vitro* cellular models and the complexity of the *in vivo* microenvironment. Even the most high-throughput screening assays utilize monolayer cell cultures, where the crucial elements of drug-biology interactions are lost (Li and Kilian, 2015). Therefore, physiologically more relevant *in vitro* human models for screening and validation of thousands of compounds are in high demand in both academic research and the pharmaceutical industry. In this context, stem-cell-based approaches using human pluripotent stem cells, including both human embryonic stem cell (ESC) and induced pluripotent stem cell (iPSC), have received great attention as effective tools for drug screening, not only for CVDs but also for other metabolic and neuronal diseases (Grskovic et al., 2011; Engle and Puppala, 2013). However, cell resources, ethical constraints, invasive extraction procedures, and expensive cell reprogramming and maintenance procedures make this type of stem cell less favorable as a practical source for drug screening (Chen et al., 2014).

In this context, human amniotic epithelial stem cells (hAESCs) possess substantial advantages over other stem cells. hAESCs are derived from term placenta after delivery and are discarded after birth. Therefore, they are readily available, do not require invasive harvesting procedures, and pose no ethical concerns. Most importantly, as hAESCs are derived from pluripotent epiblasts, they maintain ESC-like multilineage differentiation potential. Studies have shown that upon appropriate differentiation protocol, hAESCs can be differentiated into liver, pancreas and lung epithelium from endodermal origin, bone, and fat cells from mesodermal origin as well as neural cells from ectodermal origin (Miki et al., 2005, 2010; Toda et al., 2007; Parolini et al., 2008; Murphy et al., 2010; Zhou et al., 2013). Importantly, hAESCs also have cardiogenic and angiogenic differentiation potential (Miki et al., 2005; Fang et al., 2012; Wu et al., 2017; Serra et al., 2018). Furthermore, hAESCs have no tumorigenic, weak immunogenic, and strong immunomodulatory properties, and demonstrate mesenchymal stem cell (MSC)-like phenotypes as well (Miki, 2018; Yang et al., 2018; De Coppi and Atala, 2019). Thus, hAESCs provide an excellent model system for drug discovery.

At the University of Tsukuba Hospital, the Tsukuba Human Tissue Biobank Center (THB) was established in November 2013 with the aim to reserve human biospecimens to facilitate medical research (Takeuchi et al., 2016). Among a variety of cellular samples, hAESCs isolated from the donated full-term placenta are also preserved for research applications. In our previous studies, the stemness characteristics of hAESCs received from THB have been investigated (Ferdousi et al., 2019; Furuya et al., 2019). Although the primary amniotic epithelial cells were heterogenous, the hAESCs isolated from the adherent subpopulations of passaged primary cells have widely expressed stemness markers. Besides, hAESCs cultured in a 3D microenvironment as spheroids, have highly expressed the stemness-related genes compared to their 2D counterpart as well as compared to iPSCs and MSCs (Ferdousi et al., 2019). We have previously investigated the gene expression profiles of several natural compounds in hAESCs (Ferdousi et al., 2019, 2020) in an effort to screen eligible natural compounds for further in-depth investigations. Among the compounds that we have screened in hAESCs, isorhamnetin (ISO) exerts cardiac morphogenesis, and antifibrotic potential.

Isorhamnetin, also known as 3-Methylquercetin or 3’-Methoxyquercetin, is one of the most common and widely distributed plant flavonols. It has anti-inflammatory, antioxidant, antiadipogenic, and antitumor activities (Hu et al., 2015; Yang et al., 2016). We have previously reported the anti-oxidant, antiobesity, and antifibrotic effects of ISO in rodent models (Zar Kalai et al., 2013; Ganbold et al., 2019). Ganbold et al. (2019) reported that ISO could alleviate steatosis and fibrosis in a non-alcoholic steatohepatitis (NASH) mouse model by reducing the expression of liver injury marker transforming growth factor β (*Tgf*β), and the fibrogenic marker Collagen type I alpha 1 (*Col1a1*). However, little is known about the possible protective effect of ISO against cardiac fibrosis or hypertrophy (Gao et al., 2017).

This study aimed to investigate the cardioprotective potential of ISO in hAESCs through global gene expression analysis and to observe its effect on angiotensin II (AgII)-induced fibrosis and hypertrophy in the myocardium of mice.

## Materials and Methods

### *In vitro* Study

#### hAESCs Extraction and Culture

The procedure of cell isolation and cell culture have been explained elsewhere in detail (Ferdousi et al., 2019, 2020; Furuya et al., 2019). Briefly, hAESCs were obtained from the term placenta donated by mothers who underwent cesarean delivery. The amnion was washed with 200 mL of Hank’s Basic Salt Solution–Calcium and Magnesium Free (CMF-HBSS) after manual separation from the chorion and was cut into smaller pieces using a surgical scissor. The cells were maintained in placental epithelial cell basal medium (Promocell. # C-26140). The medium was changed every 2–4 days. To subculture hAESCs, the plates were first washed twice with 10 mL of PBS. Then, 3 mL of pre-digestion buffer, pre-warmed to 37°C, was added to the plate. The cells were then incubated at 37°C for 5 min. Subsequently, 5 mL of 0.05% (w/v) trypsin-EDTA, pre-warmed to 37°C, was added to the plate and incubated at 37°C for 10 min. Finally, 5 mL of Dulbecco’s modified Eagle’s medium (DMEM) was added to stop the reaction. The cell suspension was then centrifuged twice at 200 rpm for 4 min at 4°C.

#### hAESC 3D Spheroid Formation and Cell Treatment

Lipidure^TM^ (NOF Corporation, Cat. # CMS206; 400 μL) solution was placed into each well of the 3D culture plate (ElplasiaTM, Kuraray Co., Ltd., Cat # RB 500 400 NA 24) at a concentration of 50 mg in 10 mL absolute ethanol. Lipidure solution was aspirated out after 2 min. Then, the plate was dried for 3 h, 400 μL of PBS was placed in each well, and the plate was centrifuged at 2,000 × *g* for 15 min at room temperature. After discarding the PBS, the wells were washed twice with 400 μL of PBS. The plates were then stored in a cell culture incubator until use. Spheroids were formed by seeding hAESCs (8 × 10^5^ cells) in placental basal epithelial cell medium in 24-well plates. The initial culture was maintained for 24 h, and the control samples for day 0 were collected before adding the treatment. After 24 h, the medium was changed with 20 μM ISO (Sigma-Aldrich, Japan). The medium was changed every 48 h, and the cells were maintained for 10 days. Control samples were maintained in placental basal epithelial cell medium, which was also changed every 48 h.

#### hAESC RNA Extraction and Quantification

Total RNA was extracted using 1 mL of ISOGEN (Nippon Gene, Japan) following the manufacturer’s instructions. RNA quality and quantity were measured using a NanoDrop 2000 spectrophotometer (Thermo Fisher Scientific, Wilmington, DE, United States).

#### Microarray Gene Expression Profiling

Affymetrix microarray gene expression profiling was performed using GeneChip^®^ 3′ Expression Arrays and 3′ IVT PLUS Reagent Kit (Affymetrix Inc., Santa Clara, CA, United States). From 100 ng of total RNA, amplified and biotinylated complementary RNA (cRNA) was generated for each sample following the user’s manual. For hybridization, 9.4 μG cRNA was used. Human genome array strips (HG-U219) were hybridized for 16 h in a 45°C incubator, washed, and stained. Finally, imaging was performed in the GeneAtlas Fluidics and Imaging Station.

#### Microarray Data Processing and Analysis

Microarray analysis was conducted for two biological replicates of day 0 (D0) control, and three biological replicates of day 10 (D10) control and D10 ISO treated hAESCs. Microarray raw image data (.cel file) were processed and normalized following the robust multichip average (RMA) algorithm using Expression Console Software (Affymetrix, Japan). Subsequent analysis was carried out using the freely available software Transcriptome Analysis Console (TAC) version 4 (Affymetrix, Japan). Differentially expressed genes (DEGs) were characterized as fold change ≥1.5 (in linear space) and *p* < 0.05 (one-way between-subject). Gene ontology (GO) analysis was carried out using the Molecular Signatures Database (MSigDB) of Gene Set Enrichment Analysis online tool^1^ (Mootha et al., 2003; Subramanian et al., 2005) and DAVID (Database for Annotation, Visualization, and Integrated Discovery, version 6.8) (Huang et al., 2008; Sherman et al., 2008). Heat maps were generated using a web tool Heatmapper^2^ (Babicki et al., 2016).

### *In vivo* Study

#### Animal Preparation

Animal preparation: Male C57Bl/6 mice were (8 weeks of age; Japan Charles River Kanagawa, Japan) randomly assigned into three groups: control, AgII-administered group (AgII), and AgII and ISO-treated group (AgII + ISO). Mice in the AgII and AgII + ISO groups were continuously administered AgII (1,000 ng/kg/hr) with a mini-osmotic pump model (Alzet, model 2002, Cupertino, United States) for 2 weeks. Mice in the control group were infused with 0.85% saline. One week before the implantation of the mini-osmotic pumps, mice in the AgII + ISO group were administered ISO (5 mg/kg) intraperitoneally every day for 3 weeks. ISO was suspended in 0.1% dimethyl sulfoxide and 1% polypropylene glycol. Suspensions were freshly prepared and administered at a constant volume of 0.3 mL. Mice in the control and AgII groups were given the same volume of the vehicle solution (0.1% dimethyl sulfoxide, 1% polypropylene glycol, and normal saline) by intraperitoneal injection. Hearts were isolated immediately after exsanguination. Part of the left ventricles was fixed in 4% paraformaldehyde and embedded in paraffin for histological analysis. The remaining left ventricles were kept in liquid nitrogen for further analysis.

#### Echocardiography

Echocardiography was performed using a Doppler echocardiographic system (Vevo 2100; Visual Sonics, Toronto, Canada). The mice were anesthetized with 1% isoflurane. Then, parasternal short-axis and parasternal long-axis 2D-guided echocardiographic views of the left ventricle (LV) were obtained at the level of the papillary muscles. The M-mode measurements of LV posterior wall thickness (LVPWT) and interventricular septal thickness (IVST) were obtained at the end of diastole and systole. LV mass was calculated using the following formula (Marwick et al., 2015):

$$\begin{array}{l} \mathrm{LV}\,\mathrm{mass}=1.04\,\left[{\left(\mathrm{LVEDd}+\mathrm{LVPWT}+\mathrm{IVST}\right)}^{3}-{\mathrm{LVEDd}}^{3}\right]\times 0.8+0.6\\ \mathrm{LVEDd}=\mathrm{LV}\,\mathrm{end}\,\text{-}\,\mathrm{diastolic}\,\mathrm{dimension} \end{array}$$

#### Histological Examination

Left ventricle was fixed in 4% paraformaldehyde and embedded in paraffin. The epicardium of the mid-lateral wall was cut into 2 μm thick sections. All specimens were stained with Masson trichrome (MT) and hematoxylin-eosin (H-E) stain and examined under a light microscope (BZX710; Keyence, Osaka, Japan). Transmural distribution of the percentage area of fibrosis was calculated as the total area of fibrosis (defined as the amount of collagen deposition stained with the aniline blue) divided by the sum of the total tissue area. Myocyte area was measured in ∼50 cells at the site of a visible nucleus in each slide stained with H-E. The cross-sectional area of each myocyte (an average of 50 cardiomyocytes for each animal) was measured across the region corresponding to the visible nucleus in H-E stained slides.

#### Real-Time Polymerase Chain Reaction (RT-PCR)

Total RNA was extracted from ventricular tissue using an RNeasy Mini Kit (Qiagen, Valencia, CA, United States) according to the supplier’s protocol. Total RNA (1 μg) was reverse transcribed to cDNA using a High-Capacity cDNA Reverse Transcription Kit (Thermo Fisher Scientific, Inc., Waltham, MA, United States). Real-time quantitative PCR was performed with PrimeTime Gene Expression Master Mix (Integrated DNA Technologies) on the ABI Prism 7500 FAST sequence detection system (Applied Biosystems, Foster City, CA, United States). The following primers were used for the experiment: natriuretic peptide B (*Nppb*; Mm.PT.588584045.g), *Col1a1* (Mm.PT.587562513), *Tgfb1* (Mm.PT.5811254750), and *Tgfb2* (Mm.PT5814105470). Gene expression levels were normalized to the housekeeping gene, 18S rRNA (4319413E, Thermo Fisher Scientific).

#### Chemicals

ISO was synthesized from quercetin (Fujifilm Wako Pure Chemical Corp., Tokyo, Japan) following the protocol of the National Institute of Advanced Industrial Science and Technology (Kato et al., 2016) and was used for *in vitro* and *in vitro* experiments. AgII was purchased from Wako Pure Chemical Industries Ltd (Osaka, Japan).

#### Statistical Analysis

Results are expressed as the mean ± standard deviation. A one-way analysis of variance (ANOVA) followed by Tukey’s *post hoc* test was performed to examine the differences between pairs of treatment groups. Differences were considered statistically significant at *p* < 0.05. All analyses were performed using IBM SPSS version 21.0 software (IBM Co., Armonk, NY, United States).

#### Ethical Considerations

The protocol for the hAESC study was reviewed and approved by the Ethical Review Committee of the University of Tsukuba. Informed written consent was obtained from the mothers who donated the placenta after delivery.

After receiving approval from the Institutional Animal Experiment Committee of the University of Tsukuba, animal experiments were carried out in accordance with the Guide for the Care and Use of Laboratory Animals published by the US National Institutes of Health, the Regulation for Animal Experiments in the University of Tsukuba, and the Fundamental Guideline for Proper Conduct of Animal Experiments and Related Activities in Academic Research Institutions under the jurisdiction of the Ministry of Education, Culture, Sports, Science and Technology of Japan.

## Results

### ISO Regulated Cardiac Development and Fibrosis-Associated GO and KEGG Pathways

**FIGURE 1:**
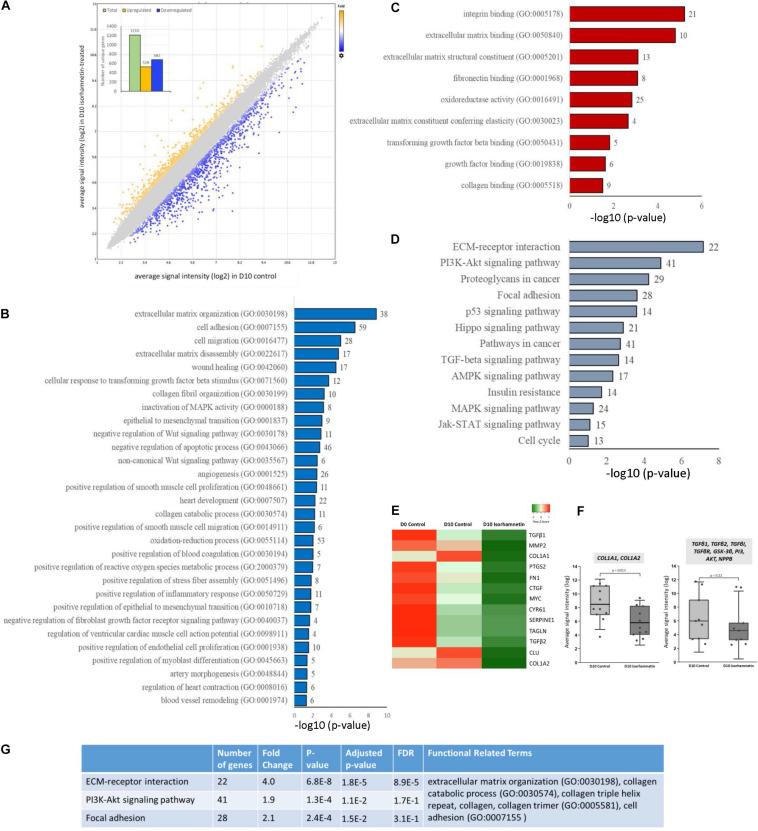
**(A)** Scatter plot showing the distribution of DEGs by fold changes. The *X*-axis corresponds to the average signal intensity (log2) of each probe ID in D10 control hAESCs, and the *Y*-axis corresponds to the average signal intensity (log2) of each probe ID in D10 Isorhamnetin-treated hAESCs. Blue dots represent the significantly downregulated and yellow dots represent the significantly upregulated DEGs (DEGs, differentially expressed genes with fold change >1.5; *p* < 0.05, one-way between-subjects ANOVA). The inset column graph shows the number of upregulated and downregulated DEGs (unique gene). Bar graphs showing the significantly enriched (*p* < 0.05; modified Fisher’s exact test) **(B)** biological processes, **(C)** molecular functions, and **(D)** KEGG pathways by the DEGs between D10 isorhamnetin-treated and D10 control hAESCs. The *X*-axis corresponds to the –log10 *p*-value. The number of DEGs is presented at the outer end of each bar. **(E)** Heatmap showing the expression intensity of selected cardiac fibrosis-associated genes. Heatmap was generated using an online tool Heatmapper (http://www.heatmapper.ca/). **(F)** Boxplots showing the comparison of expression intensity of all probe IDs of selected genes between D10 control and D10 isorhamnetin-treated hAESCs. Box ranges from 25th to 75th percentile, the line in the middle represents the median value, and the error bar represents the standard deviation (SD). Significance was computed by *t*-test for linear distributions and Mann-Whitney *U* test for non-linear distributions. **(G)** Table showing the most significantly enriched annotation cluster computed for KEGG pathways and biological processes. The enrichment score for this fibrosis-associated cluster was 4.89 (measurement criteria: Similarity threshold = 0.5, multiple linkage threshold = 0.5, and enrichment thresholds = 1.0).

A total of 1210 unique genes (after excluding duplicate probe IDs) were differentially expressed (fold change >1.5; *p* < 0.05) in ISO-treated hAESCs compared to untreated controls at D10. Among them, 528 genes were significantly upregulated, whereas 682 genes were downregulated (Figure 1A). Compared to D0 control, a total of 3449 and 3884 genes were significantly (fold change > 2; *p* < 0.05) regulated in D10 control and D10 ISO-treated hAESCs, respectively (Supplementary Figure 1). Compared to undifferentiated hAESCs at D0, control hAESCs at D10 regulated epithelial-mesenchymal transition (EMT), cell cycle, cell division, autophagy, and apoptosis-associated GO (Supplementary Figure 1), whereas ISO-treated hAESCs regulated anti-inflammatory and TGFβ-related biological functions as well as EMT. The differentiation potential was toward myogenesis (Supplementary Figure 1). We have previously reported that the undifferentiated hAESCs expressed several stem cell markers, and the 3D hAESC spheroids showed significantly higher expression of stemness-related genes than their 2D counterparts.

In this article, we will mainly explain the GO enriched by DEGs between D10 ISO-treated and untreated control hAESCs. Significantly enriched biological processes included ECM organization, TGFβ and collagen-related processes, and anti-inflammatory functions. Both canonical and non-canonical Wnt signaling pathways and MAPK pathways were regulated. Cell adhesion, cell migration, wound healing, EMT, and apoptosis were also significantly regulated. Additionally, several cardiovascular development-associated pathways were also significantly regulated, which included but were not limited to heart development, angiogenesis, smooth muscle cell proliferation and migration, endothelial cell proliferation, artery morphogenesis, and blood vessel remodeling. Furthermore, ventricular cardiac muscle cell action potential, heart contraction, and myoblast differentiation-associated biological processes were significantly enriched (Figure 1B). Similarly, significantly enriched molecular functions included ECM structural constituents, elasticity, and binding as well as collagen binding, TGFβ binding, and fibronectin binding (Figure 1C). The most significant KEGG pathways were ECM-receptor interaction, focal adhesion, and PI3K-Akt, p53, TGFβ, MAPK, AMPK, and Jak-STAT pathways (Figure 1D). Heat map (Figure 1E) and boxplots (Figure 1F) show that fibrosis-associated genes had decreased expression profiles in ISO-treated hAESCs compared to untreated control cells. Finally, hierarchical cluster analysis of KEGG pathways and biological processes identified 33 clusters. The most significant cluster involved three KEGG pathways: ECM-receptor interaction, PI3K-Akt signaling pathway, and focal adhesion (enrichment score 4.89, Figure 1G). Genes involved in this cluster and their fold changes are listed in Supplementary Table 1.

### ISO Abrogated AgII-Induced Ventricular Hypertrophy *in vivo*

**FIGURE 2:**
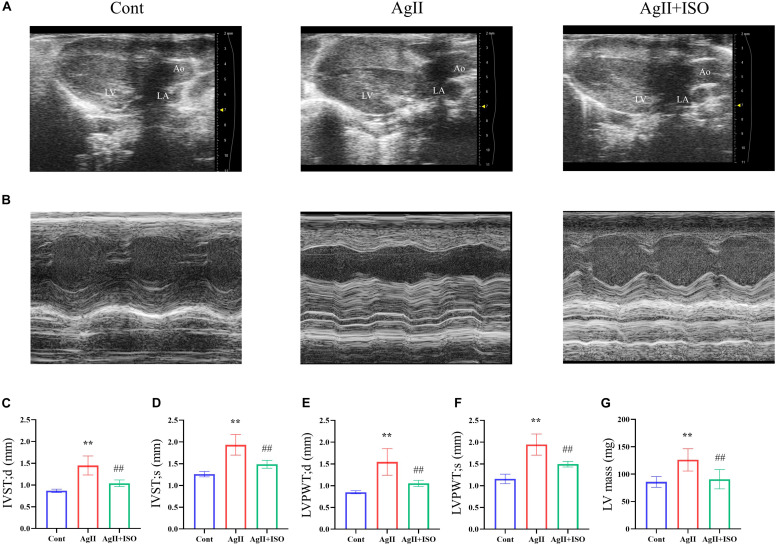
**(A,B)** Representative B-mode and M-mode echocardiograms of 11-week-old mice from the three groups. **(C–G)** Echocardiographic parameters, including IVS-s, IVS-d, LVPW-s, LVPW-d, and LV mass. Data were evaluated at 11 weeks in the Cont, AgII, and AgII + ISO groups (*n* = 5–7 per group). **P < 0.01 vs. Cont. ^##^*P* < 0.01 vs. AgII + ISO. IVST-s, LV interventricular septal thicknesses in systole; IVS-d, LV interventricular septal thickness in diastole; LVPWT-s, LV posterior wall thickness in systole; LVPWT-d, LV posterior wall thickness in diastole; LV, left ventricle.

To investigate the protective effect of ISO on abnormal cardiac structure induced by AgII, two-dimensional echocardiography was performed (Figures 2A,B). The echocardiography results indicate that the interventricular septum (IVS) and left ventricular posterior wall (LVPW) thickness, at both systole and diastole, were significantly increased in AgII-induced mice (*p* < 0.01 vs. control), while the increased IVS and LVPW thickness were significantly decreased by ISO treatment (Figures 2C–F). In addition, B-mode images indicated that the walls of LV were enlarged by AgII and were restored by ISO treatment. Moreover, LV mass was significantly increased in the AgII-induced group. ISO could abrogate the AgII-induced increase in LV mass (Figure 2G).

### ISO Reversed AgII-Induced Morphological Abnormalities in Cardiac Tissue *in vivo*

**FIGURE 3:**
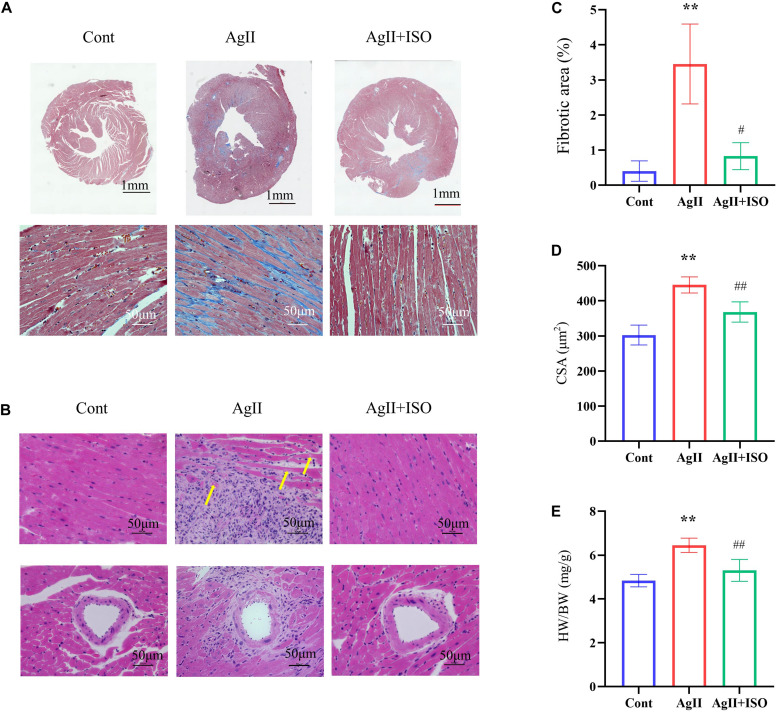
Histological analyses of MT staining **(A)** and H-E staining **(B)** of each group of mice at 11 weeks. The yellow arrows indicate cytoplasmic vacuolization of cardiomyocytes. **(C)** The fibrotic area to the total area (*n* = 3 per group). **(D)** The results of the quantitative analysis of the cross-sectional area (CSA). **(E)** The statistical results of HW/BW (*n* = 7 per group). *P < 0.05, **P < 0.01 vs. Cont. ^#^*P* < 0.05, ^##^*P* < 0.01 vs. AgII + ISO.

MT staining images showed that ISO significantly prevented the myocardial fibrotic responses induced by AgII (Figure 3A). Compared to the control group, H-E images showed severe disorganization of myofibrillar arrays, and cytoplasmic vacuolization and infiltration with neutrophil granulocytes in AgII-treated mice. ISO pretreatment remarkably reduced the pathological abnormalities (Figure 3B). The percentage of total fibrotic area was significantly increased in AgII-induced group, whereas ISO treatment could significantly reduce the percentage of fibrosed area (Figure 3C). Furthermore, ISO significantly reduced the AgII-mediated increase in cardiomyocyte size (measured as cross-sectional area; CSA) (Figure 3D). The ratio of heart weight/body weight (HW/BW) was significantly increased by stimulation with AgII (*p* < 0.01 vs. control), while ISO significantly reduced the increase in HW/BW (Figure 3E).

### ISO Prevented AgII-Induced Expression of Inflammatory and Fibrogenic Markers in Mouse Cardiac Tissue *in vivo*

To confirm the preventive effects of ISO on fibrogenic and hypertrophy markers observed in microarray analysis of hAESCs, we conducted RT-PCR in ventricular tissue to quantify the expression of *Tgf*β*1*, *Tgf*β*2*, *Col1a1*, and *Nppb*.

**FIGURE 4:**
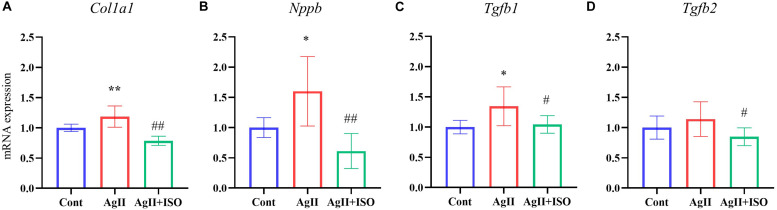
**(A–D)** The relative expression levels of *Col1a1*, *Nppb*, *Tgfb1*, and *Tgfb2* evaluated by RT-PCR for the three groups (*n* = 6–8 per group). The transcript levels were normalized to 18s and presented as a relative value. *P < 0.05, **P < 0.01 vs. Cont. ^#^*P* < 0.05, ^##^*P* < 0.01 vs. AgII + ISO.

*Tgf*β*1*plays an important role in the development of myocardial fibrosis by promoting the production and deposition of collagens (Lijnen et al., 2000). AgII administration has been reported to increase the expression of *Tgf*β*1 in vivo*, leading to cardiac fibrosis. In addition, overexpression of *Tgf*β*1* induces the mRNA expression of collagen type I (*Col1A1*) in rodent cardiac fibroblasts. We found that ISO significantly attenuated AgII-induced expression of *Tgf*β*1*, *Tgf*β*2*, and *Col1a1* (Figure 4).

Furthermore, pretreatment with ISO significantly decreased AgII-induced overexpression of *Nppb*. It encodes the brain natriuretic peptide (BNP) hormone, which is an important biomarker in clinical cardiology. In response to increased cardiac stress, fibrosis, and hypertrophy, BNP is strongly upregulated in the ventricular cardiac muscle.

## Discussion

In this study, we showed that ISO regulated cardiac fibrosis-related GO and KEGG pathways in hAESCs. We then successfully translated the *in vitro* findings in the *in vivo* model. ISO suppressed myocardial hypertrophy and fibrosis induced by AgII in mice ventricles. In short, the present study demonstrates that ISO could be a valuable pharmaceutical or dietary supplement.

Both pathological fibrosis and hypertrophy of the heart can induce heart failure and other serious cardiac diseases. These are also independent risk factors for cardiac morbidity and mortality. Conventional drugs, including angiotensin-converting enzyme inhibitors, β-blockers, statins, and aldosterone antagonists, are used for the inhibition of cardiac fibrosis and associated complications, however, their effects are secondary to the alleviation of cardiac dysfunction rather than directly targeting cardiac hypertrophy or fibrosis (Zannad et al., 2000; Bauersachs et al., 2001; Klapholz, 2009). Therefore, the discovery of novel compounds with profibrotic potential is urgently needed. Considering the inefficient translation of CVD drugs from hundreds of chemical libraries, a stemcell-based drug screening approach would be most appropriate. In this context, hAESC, a perinatal stem cell that possesses both ESC-like pluripotent potential and adult stem cell-like immunomodulatory properties, has drawn great attention from researchers worldwide because of their considerable advantages over other stem cells. hAESCs are readily available, have minimum ethical concerns, and are cost-effective. A number of studies have demonstrated the multilineage differentiation potential of hAESCs (Miki et al., 2005, 2010; Toda et al., 2007; Parolini et al., 2008; Murphy et al., 2010; Zhou et al., 2013). Notably, hAESCs have also been reported to differentiate into cardiomyocytes (Miki et al., 2005; Fang et al., 2012) and endothelial cells (Wu et al., 2017; Serra et al., 2018). Therefore, hAESC is a good alternative tool for screening potential drugs for CVD.

One of the major obstacles of translating hAESCs into the clinic is the heterogeneity in primary amnion epithelial cell population and discrepancies in their cell surface profiling (Centurione et al., 2018) that can be primarily attributed to isolation protocols, gestational age, passage number, and epithelial to mesenchymal transition (Ghamari et al., 2020). However, hAESCs isolated from the adherent subpopulations of passaged primary cells, and cultured in 3D environment may successfully overcome these limitations and may express a higher level of stemness properties (Ferdousi et al., 2019; Furuya et al., 2019). Additionally, protocol for immortalized human amniotic epithelial cells with high expressions of stem cell markers has been reported (Zhou et al., 2013).

In the present study, we have evaluated the cardioprotective potential of ISO in hAESCs. Previously, we have reported that ISO, a flavonol, alleviates hepatic fibrosis in NASH model mice (Ganbold et al., 2019). Although several flavonols have been reported to improve cardiac dysfunction and fibrosis (Suzuki et al., 2007; Li et al., 2013; Geetha et al., 2014; Guo et al., 2015; Zhang et al., 2018), very little is known about the effect of ISO on cardiac hypertrophy and fibrosis. Microarray analysis of ISO-treated hAESCs revealed that ISO has strong antifibrotic effects as well as anti-inflammatory and antioxidative functions. Additionally, ISO showed myogenic and angiogenic differentiation potential in hAESCs. Interestingly, GO analysis showed that ISO targeted similar biological functions in hAESCs that were reported to be regulated in the early stage of AgII-induced cardiac remodeling in a mouse model (Dang et al., 2015). We also found that ISO significantly regulated both canonical and non-canonical Wnt pathways, TGFβ, and MAPK pathways, all of which are cross-regulated in the progression of profibrotic changes in the heart (Działo et al., 2018).

Next, we evaluated the effect of ISO on AgII-induced progression of myocardial fibrosis and hypertrophy *in vivo*. We found that ISO could effectively attenuate the expression levels of *Tgf*β*1* induced by AgII. TGFβ1, a potent fibrogenic cytokine, plays an important role in the synthesis of collagen, fibronectin, or other intercellular substances, which leads to enhanced expression levels of collagen type I. Much evidence suggests that TGFβ1 overexpression is a common pathway for various pathological factors that cause myocardial fibrosis. TGFβ1 has also been shown to prevent or reverse organ fibrosis via the TGFβ/Smad signaling system.

Myocardial collagen consists mainly of type I and type III collagen. Type I collagen (Lu et al., 2018) attenuates the network for cardiac muscle cells and strengthens myocardial tissue. Although collagen initially has a structural role in preventing ventricular rupture, an increase in the ratio of type I to type III collagen has been reported in hearts with chronic congestive heart failure, which ultimately leads to myocardial death. The present study showed that the expression of *Col1a1* enhanced by AgII was significantly reduced by ISO treatment. We hypothesized that ISO could reverse the expression levels of collagen by attenuating TGFβ1 pathways, thereby suppressing the development of fibrosis.

Furthermore, this study shows that ISO significantly suppressed *Nppb* gene expression. *Nppb* encodes BNP hormone, which is an important biomarker in clinical cardiology (Diamandis et al., 2014). In response to increased cardiac stress, fibrosis, and hypertrophy, BNP is strongly upregulated in the ventricular cardiac muscle (Kerkelä et al., 2015). BNP inhibits renin secretion, thereby inhibiting the renin-angiotensin-aldosterone system. Therefore, our findings indicate that ISO has the effects on cardiac hypertrophy and fibrosis.

It has been reported that ISO ameliorates cardiac hypertrophy and fibrosis induced by aortic banding (Gao et al., 2017), however, there has been no report on the effect of ISO on AgII loading. Although the mechanism of angiotensin-induced cardiac disease has largely been clarified, researchers are still searching for effective drugs. A commonly used animal model is the subcutaneous infusion of AgII into mice. Osmotic pumps are designed to continuously deliver compounds to small animals at a constant rate. This technique is commonly used to induce AF, aortic aneurysm, and hypertension, leading to serious heart disease or sudden death (Wu et al., 2019). AgII 1,000 ng/kg/min is commonly used. Based on this background, we conducted this experiment with reference to this dose. AgII, a vasoactive peptide, is a key regulator of hypertension, inflammatory response, hypertrophy, and fibrosis through the regulation of multiple signaling pathways, such as NF-κB and TGFβ1/Smad (Shang et al., 2008). Our results indicated that chronic AgII infusion remarkably induced left ventricular wall extension, dilation, pathological hypertrophy, fibrosis, and macrophage infiltration, and that ISO completely eliminated these abnormalities induced by AgII. ISO significantly suppressed fibrosis in the interstitial myocardium and around blood vessels showed in MT staining. Furthermore, ISO prevented the living cells surrounded by dead cells that eventually became oxygen-deficient, leading to heart failure and sudden death.

## Conclusion

Our evidence suggests that hAESC has important implications as an alternative tool for the screening and development of drugs and dietary supplements in future. Our results also demonstrate that ISO could effectively suppress hypertrophy and fibrosis induced by AgII in cardiac tissue by regulating TGFβ pathways. ISO could have beneficial effects on potential clinical consequences of CVDs by regulating the activity of the renin-angiotensin system.

## Data Availability Statement

All data generated or analyzed during this study are included in this article and its Supplementary Information. Microarray data are deposited in the Gene Expression Omnibus (GEO) under accession number: GSE153149 (https://www.ncbi.nlm.nih.gov/geo/query/acc.cgi?acc=GSE153149).

## Ethics Statement

The protocol for the hAESC study was reviewed and approved by the Ethical Review Committee of the University of Tsukuba. Informed written consent was obtained from the mothers who donated the placenta after delivery.

After receiving approval from the Institutional Animal Experiment Committee of the University of Tsukuba, animal experiments were carried out in accordance with the Guide for the Care and Use of Laboratory Animals published by the US National Institutes of Health, the Regulation for Animal Experiments in the University of Tsukuba, and the Fundamental Guideline for Proper Conduct of Animal Experiments and Related Activities in Academic Research Institutions under the jurisdiction of the Ministry of Education, Culture, Sports, Science and Technology of Japan.

## Author Contributions

KA: investigation. KA and FF: formal analysis, data curation, software, visualization, and writing-original draft. DX: methodology, validation, supervision, and resources. KT: investigation, validation, and resources. HI: conceptualization, methodology, resources, supervision, project administration, funding acquisition, and writing-review and editing. All authors made substantial contributions to this article and approved the final version of the article.

## Conflict of Interest

The authors declare that the research was conducted in the absence of any commercial or financial relationships that could be construed as a potential conflict of interest.

## Footnotes

1 https://software.broadinstitute.org/gsea/index.jsp

2 http://www.heatmapper.ca/

## References

[B1] Babicki S. Arndt D. Marcu A. Liang Y. Grant J. R. Maciejewski A. (2016). Heatmapper: web-enabled heat mapping for all. *Nucleic Acids Res.* 44 W147–W153. 27190236 10.1093/nar/gkw419 PMC4987948

[B2] Bang C. N. Soliman E. Z. Simpson L. M. Davis B. R. Devereux R. B. Okin P. M. (2017). Electrocardiographic left ventricular hypertrophy predicts cardiovascular morbidity and mortality in hypertensive patients: the ALLHAT Study. *Am. J. Hypertens.* 30 914–922. 10.1093/ajh/hpx067 28430947 PMC5861536

[B3] Bauersachs J. Galuppo P. Fraccarollo D. Christ M. Ertl G. (2001). Improvement of left ventricular remodeling and function by hydroxymethylglutaryl coenzyme a reductase inhibition with cerivastatin in rats with heart failure after myocardial infarction. *Circulation* 104 982–985. 11524389 10.1161/hc3401.095946

[B4] Centurione L. Passaretta F. Centurione M. A. De Munari S. Vertua E. Silini A. (2018). Mapping of the human placenta: experimental evidence of amniotic epithelial cell heterogeneity. *Cell Transpl.* 27 12–22. 10.1177/0963689717725078 PMC6434477 29562779

[B5] Chen K. G. Mallon B. S. McKay R. D. G. Robey P. G. (2014). Human pluripotent stem cell culture: considerations for maintenance, expansion, and therapeutics. *Cell Stem Cell* 14 13–26. 10.1016/j.stem.2013.12.005 24388173 PMC3915741

[B6] Dang M.-Q. Zhao X.-C. Lai S. Wang X. Wang L. Zhang Y.-L. (2015). Gene expression profile in the early stage of angiotensin II-induced cardiac remodeling: a time series microarray study in a mouse model. *Cell. Physiol. Biochem.* 35 467–476. 25613478 10.1159/000369712

[B7] De Coppi P. Atala A. (2019). “Stem cells from the amnion,” in *Principles of Regenerative Medicine*, 3rd Edn, eds Atala A. Lanza R. Mikos A. G. Nerem R. (Boston: Academic Press), 133–148.

[B8] Diamandis E. P. Maisel A. Jaffe A. S. Clerico A. (2014). Q & A: natriuretic peptides in heart failure. *Clin. Chem.* 60 1040–1046. 24700774 10.1373/clinchem.2014.223057

[B9] Działo E. Tkacz K. Błyszczuk P. (2018). Crosstalk between TGF-β and WNT signalling pathways during cardiac fibrogenesis. *Acta Biochim. Polonica* 65 341–349. 10.18388/abp.2018_2635 30040870

[B10] Engle S. J. Puppala D. (2013). Integrating human pluripotent stem cells into drug development. *Cell Stem Cell* 12 669–677. 23746976 10.1016/j.stem.2013.05.011

[B11] Fang C.-H. Jin J. Joe J.-H. Song Y.-S. So B.-I. Lim S. M. (2012). In vivo differentiation of human amniotic epithelial cells into cardiomyocyte-like cells and cell transplantation effect on myocardial infarction in rats: comparison with cord blood and adipose tissue-derived mesenchymal stem cells. *Cell Transpl.* 21 1687–1696. 10.3727/096368912X653039 22776022

[B12] Ferdousi F. Kondo S. Sasaki K. Uchida Y. Ohkohchi N. Zheng Y.-W. (2020). Microarray analysis of verbenalin-treated human amniotic epithelial cells reveals therapeutic potential for Alzheimer’s disease. *Aging* 12:5516. 10.18632/aging.102985 PMC7138585 32224504

[B13] Ferdousi F. Sasaki K. Ohkohchi N. Zheng Y.-W. Isoda H. (2019). Exploring the potential role of rosmarinic acid in neuronal differentiation of human amnion epithelial cells by microarray gene expression profiling. *Front. Neurosci.* 13:779. 10.3389/fnins.2019.00779 31396047 PMC6667736

[B14] Furuya K. Zheng Y.-W. Sako D. Iwasaki K. Zheng D.-X. Ge J.-Y. (2019). Enhanced hepatic differentiation in the subpopulation of human amniotic stem cells under 3D multicellular microenvironment. *World J. Stem Cells* 11:705. 10.4252/wjsc.v11.i9.705 PMC6789189 31616545

[B15] Ganbold M. Owada Y. Ozawa Y. Shimamoto Y. Ferdousi F. Tominaga K. (2019). Isorhamnetin alleviates steatosis and fibrosis in mice with nonalcoholic steatohepatitis. *Sci. Rep.* 9:16210. 10.1038/s41598-019-52736-y 31700054 PMC6838085

[B16] Gao L. Yao R. Liu Y. Wang Z. Huang Z. Du B. (2017). Isorhamnetin protects against cardiac hypertrophy through blocking PI3K–AKT pathway. *Mol. Cell. Biochem.* 429 167–177. 28176246 10.1007/s11010-017-2944-x

[B17] Geetha R. Yogalakshmi B. Sreeja S. Bhavani K. Anuradha C. V. (2014). Troxerutin suppresses lipid abnormalities in the heart of high-fat–high-fructose diet-fed mice. *Mol. Cell. Biochem.* 387 123–134. 24173620 10.1007/s11010-013-1877-2

[B18] Ghamari S.-H. Abbasi-Kangevari M. Tayebi T. Bahrami S. Niknejad H. (2020). The bottlenecks in translating placenta-derived amniotic epithelial and mesenchymal stromal cells into the clinic: current discrepancies in marker reports. *Front. Bioeng. Biotechnol.* 8:180. 10.3389/fbioe.2020.00180 32232037 PMC7083014

[B19] Grskovic M. Javaherian A. Strulovici B. Daley G. Q. (2011). Induced pluripotent stem cells—opportunities for disease modelling and drug discovery. *Nat. Rev. Drug Discov.* 10 915–929. 22076509 10.1038/nrd3577

[B20] Guo H. Zhang X. Cui Y. Zhou H. Xu D. Shan T. (2015). Taxifolin protects against cardiac hypertrophy and fibrosis during biomechanical stress of pressure overload. *Toxicol. Appl. Pharmacol.* 287 168–177. 26051872 10.1016/j.taap.2015.06.002

[B21] Hu S. Huang L. Meng L. Sun H. Zhang W. Xu Y. (2015). Isorhamnetin inhibits cell proliferation and induces apoptosis in breast cancer via Akt and mitogen-activated protein kinase kinase signaling pathways. *Mol. Med. Rep.* 12 6745–6751. 26502751 10.3892/mmr.2015.4269 PMC4626180

[B22] Huang D. W. Sherman B. T. Lempicki R. A. (2008). Systematic and integrative analysis of large gene lists using DAVID bioinformatics resources. *Nat. Protoc.* 4:44. 10.1038/nprot.2008.211 19131956

[B23] Izawa H. Murohara T. Nagata K. Isobe S. Asano H. Amano T. (2005). Mineralocorticoid receptor antagonism ameliorates left ventricular diastolic dysfunction and myocardial fibrosis in mildly symptomatic patients with idiopathic dilated cardiomyopathy: a pilot study. *Circulation* 112 2940–2945. 10.1161/CIRCULATIONAHA.105.571653 16275882

[B24] Jin L. Sun S. Ryu Y. Piao Z. H. Liu B. Choi S. Y. (2018). Gallic acid improves cardiac dysfunction and fibrosis in pressure overload-induced heart failure. *Sci. Rep.* 8 1–11. 10.1038/s41598-018-27599-4 29915390 PMC6006337

[B25] Kai H. Mori T. Tokuda K. Takayama N. Tahara N. Takemiya K. (2006). Pressure overload-induced transient oxidative stress mediates perivascular inflammation and cardiac fibrosis through angiotensin II. *Hypertens. Res.* 29 711–718. 10.1291/hypres.29.711 17249527

[B26] Kato K. Ninomiya M. Tanaka K. Koketsu M. (2016). Effects of functional groups and sugar composition of quercetin derivatives on their radical scavenging properties. *J. Nat. Prod.* 79 1808–1814. 10.1021/acs.jnatprod.6b00274 27314621

[B27] Kerkelä R. Ulvila J. Magga J. (2015). Natriuretic peptides in the regulation of cardiovascular physiology and metabolic events. *J. Am. Heart Assoc.* 4 1–13. 10.1161/JAHA.115.002423 26508744 PMC4845118

[B28] Klapholz M. (2009). *β-Blocker Use for. (the)Stages of Heart Failure*. Amsterdam: Elsevier, 718–729. 10.4065/84.8.718

[B29] Li M. Jiang Y. Jing W. Sun B. Miao C. Ren L. (2013). Quercetin provides greater cardioprotective effect than its glycoside derivative rutin on isoproterenol-induced cardiac fibrosis in the rat. *Can. J. Physiol. Pharmacol.* 91 951–959. 10.1139/cjpp-2012-0432 24117263

[B30] Li Y. Kilian K. A. (2015). Bridging the gap: from 2D cell culture to 3D microengineered extracellular matrices. *Adv. Healthc. Mater.* 4 2780–2796. 10.1002/adhm.201500427 26592366 PMC4780579

[B31] Lijnen P. J. Petrov V. V. Fagard R. H. (2000). Induction of cardiac fibrosis by transforming growth factor-β1. *Mol. Genet. Metab.* 71 418–435. 10.1006/mgme.2000.3032 11001836

[B32] Lu Y. Kamel-El Sayed S. A. Wang K. Tiede-Lewis L. A. M. Grillo M. A. Veno P. A. (2018). Live imaging of type I collagen assembly dynamics in osteoblasts stably expressing GFP and mCherry-tagged collagen constructs. *J. Bone Miner. Res.* 33 1166–1182. 10.1002/jbmr.3409 29461659 PMC6425932

[B33] Marwick T. H. Gillebert T. C. Aurigemma G. Chirinos J. Derumeaux G. Galderisi M. (2015). Recommendations on the use of echocardiography in adult hypertension: a report from the european association of cardiovascular imaging (EACVI) and the american society of echocardiography (ASE). *J. Am. Soc. Echocardiogr.* 28 727–754. 10.1016/j.echo.2015.05.002 26140936

[B34] Miki T. (2018). Stem cell characteristics and the therapeutic potential of amniotic epithelial cells. *Am. J. Reprod. Immunol.* 80:e13003. 10.1111/aji.13003 29956869

[B35] Miki T. Lehmann T. Cai H. Stolz D. B. Strom S. C. (2005). Stem cell characteristics of amniotic epithelial cells. *Stem cells* 23 1549–1559. 10.1634/stemcells.2004-0357 16081662

[B36] Miki T. Marongiu F. Dorko K. Ellis E. C. Strom S. C. (2010). Isolation of amniotic epithelial stem cells. *Curr. Protoc. Stem Cell Biol.* 12 1E. 3.1–1E. 3.10. 10.1002/9780470151808.sc01e03s12 20049689

[B37] Mootha V. K. Lindgren C. M. Eriksson K.-F. Subramanian A. Sihag S. Lehar J. (2003). PGC-1α-responsive genes involved in oxidative phosphorylation are coordinately downregulated in human diabetes. *Nat. Genet.* 34:267. 10.1038/ng1180 12808457

[B38] Murphy S. Rosli S. Acharya R. Mathias L. Lim R. Wallace E. (2010). Amnion epithelial cell isolation and characterization for clinical use. *Curr. Prot. Stem Cell Biol.* 13 1E. 6.1–1E. 6.25. 10.1002/9780470151808.sc01e06s13 20373516

[B39] Parolini O. Alviano F. Bagnara G. P. Bilic G. Bühring H. J. Evangelista M. (2008). Concise review: isolation and characterization of cells from human term placenta: outcome of the first international workshop on placenta derived stem cells. *Stem Cells* 26 300–311. 10.1634/stemcells.2007-0594 17975221

[B40] Pott A. Rottbauer W. Just S. (2020). Streamlining drug discovery assays for cardiovascular disease using zebrafish. *Expert Opin. Drug Discov.* 15 27–37. 10.1080/17460441.2020.1671351 31570020

[B41] Serra M. Marongiu M. Contini A. Miki T. Cadoni E. Laconi E. (2018). Evidence of amniotic epithelial cell differentiation toward hepatic sinusoidal endothelial cells. *Cell Transpl.* 27 23–30. 10.1177/0963689717727541 29562778 PMC6434484

[B42] Shang L. L. Sanyal S. Pfahnl A. E. Jiao Z. Allen J. Liu H. (2008). NF-κB-dependent transcriptional regulation of the cardiac scn5a sodium channel by angiotensin II. *Am. J. Physiol. Cell Physiol.* 294 372–379. 10.1152/ajpcell.00186.2007 18032528 PMC3150844

[B43] Sherman B. T. Huang D. W. Lempicki R. A. (2008). Bioinformatics enrichment tools: paths toward the comprehensive functional analysis of large gene lists. *Nucleic Acids Res.* 37 1–13. 10.1093/nar/gkn923 19033363 PMC2615629

[B44] Subramanian A. Tamayo P. Mootha V. K. Mukherjee S. Ebert B. L. Gillette M. A. (2005). Gene set enrichment analysis: a knowledge-based approach for interpreting genome-wide expression profiles. *Proc. Natl. Acad. Sci. U.S.A.* 102 15545–15550. 10.1073/pnas.0506580102 16199517 PMC1239896

[B45] Suzuki J. I Ogawa M. Futamatsu H. Kosuge H. Sagesaka Y. M. Isobe M. (2007). Tea catechins improve left ventricular dysfunction, suppress myocardial inflammation and fibrosis, and alter cytokine expression in rat autoimmune myocarditis. *Eur. J. Heart Fail.* 9 152–159. 10.1016/j.ejheart.2006.05.007 16829193

[B46] Takeuchi T. Noguchi M. Kawakami Y. Ohkohchi N. (2016). Use of human biospecimen resources for drug discovery: approach of tsukuba human tissue biobank center. *Regul. Sci. Med. Prod.* 6 57–63. 10.14982/rsmp.6.57

[B47] Toda A. Okabe M. Yoshida T. Nikaido T. (2007). The potential of amniotic membrane/amnion-derived cells for regeneration of various tissues. *J. Pharmacol. Sci.* 105 215–228. 17986813 10.1254/jphs.cr0070034

[B48] Travers J. G. Kamal F. A. Robbins J. Yutzey K. E. Blaxall B. C. (2016). Cardiac fibrosis: the fibroblast awakens. *Circ. Res.* 118 1021–1040. 10.1161/CIRCRESAHA.115.306565 26987915 PMC4800485

[B49] Weber K. T. Sun Y. Díez J. (2008). Fibrosis: a living tissue and the infarcted heart. *J. Am. Coll. Cardiol.* 52 2029–2031. 10.1016/j.jacc.2008.09.012 19055995

[B50] Wu Q. Fang T. Lang H. Chen M. Shi P. Pang X. (2017). Comparison of the proliferation, migration and angiogenic properties of human amniotic epithelial and mesenchymal stem cells and their effects on endothelial cells. *Int. J. Mol. Med.* 39 918–926. 10.3892/ijmm.2017.2897 28259958 PMC5360425

[B51] Wu Y. X. Han X. Chen C. Zou L. X. Dong Z. C. Zhang Y. L. (2019). Time series gene expression profiling and temporal regulatory pathway analysis of angiotensin II induced atrial fibrillation in mice. *Front. Physiol.* 10 1–10. 10.3389/fphys.2019.00597 31191333 PMC6548816

[B52] Yang J. H. Kim S. C. Kim K. M. Jang C. H. Cho S. S. Kim S. J. (2016). *Isorhamnetin attenuates* liver fibrosis by inhibiting TGF-β/Smad signaling and relieving oxidative stress. *Eur. J. Pharmacol.* 783 92–102. 10.1016/j.ejphar.2016.04.042 27151496

[B53] Yang P.-J. Yuan W.-X. Liu J. Li J.-Y. Tan B. Qiu C. (2018). Biological characterization of human amniotic epithelial cells in a serum-free system and their safety evaluation. *Acta Pharmacol. Sin.* 39 1305–1316. 10.1038/aps.2018.22 29565036 PMC6289351

[B54] Yue L. Xie J. Nattel S. (2011). Molecular determinants of cardiac fibroblast electrical function and therapeutic implications for atrial fibrillation. *Cardiovasc. Res.* 89 744–753. 10.1093/cvr/cvq329 20962103 PMC3039247

[B55] Zannad F. Alla F. Dousset B. Perez A. Pitt B. (2000). Limitation of excessive extracellular matrix turnover may contribute to survival benefit of spironolactone therapy in patients with congestive heart failure: insights from the randomized aldactone evaluation study (RALES). *Circulation* 102 2700–2706. 10.1161/01.CIR.102.22.2700 11094035

[B56] Zar Kalai F. Han J. Ksouri R. El Omri A. Abdelly C. Isoda H. (2013). Antiobesity effects of an edible halophyte *Nitraria retusa* Forssk in 3T3-L1 preadipocyte differentiation and in C57B6J/L mice fed a high fat diet-induced obesity. *Evid. Based Complement. Altern. Med.* 2013:368658. 10.1155/2013/368658 24367387 PMC3866713

[B57] Zhang N. Wei W.-Y. Li L.-L. Hu C. Tang Q.-Z. (2018). Therapeutic potential of polyphenols in cardiac fibrosis. *Front. Pharmacol.* 9:122. 10.3389/fphar.2018.00122 29497382 PMC5818417

[B58] Zhou K. Koike C. Yoshida T. Okabe M. Fathy M. Kyo S. (2013). Establishment and characterization of immortalized human amniotic epithelial cells. *Cell. Reprogram.* 15 55–67. 10.1089/cell.2012.0021 23298399 PMC3567704

