# Supplementary material for: Effects of Isorhamnetin in Human Amniotic Epithelial Stem Cells in vitro and Its Cardioprotective Effects in vivo
Source: Front Cell Dev Biol. 2020 Sep 29;8:578197. doi: 10.3389/fcell.2020.578197 (PMC7552739; doi:10.3389/fcell.2020.578197)
Supplement: Supplementary file 1 [file Data_Sheet_1.PDF]

frontiers

Supplementary Material

(A)

(B)

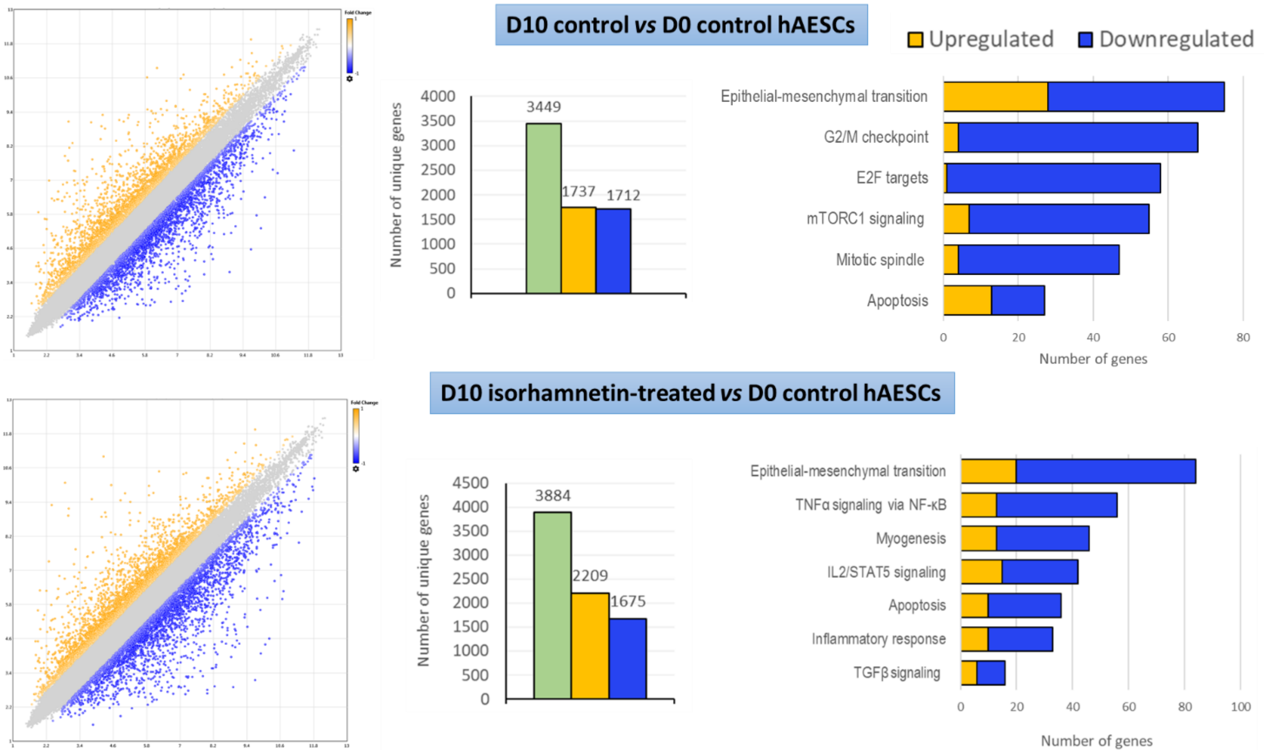

Supplementary Figure 1. (A) Scatter plot showing the distribution of DEGs in D10 control *vs* D0 control hAESCs (Fold change > 2; $p < 0.05$). The X-axis corresponds to the average signal intensity (log2) of each probe ID in D0 control hAESCs, and the Y-axis corresponds to the average signal intensity (log2) of each probe ID in D10 control hAESCs. Blue dots represent the significantly downregulated and yellow dots represent the significantly upregulated DEGs. Bar graphs showing the number of unique genes and significantly enriched biological processes. **(B)** Scatter plot showing the distribution of DEGs in D10 isorhamnetin-treated *vs* D0 control hAESCs (Fold change > 2; $p < 0.05$). Bar graph showing the number of unique genes and significantly enriched biological processes.

Supplementary Table 1. List of genes involved in the most significantly enriched cluster and their fold changes

**Gene name**	**Fold Change (D10 isorhamnetin vs D10 control)**	**p-value**
Collagen type I alpha 1 chain(COL1A1)	-8.62	1.28E-10
Thrombospondin 1(THBS1)	-8.33	3.38E-12
Collagen type I alpha 2 chain(COL1A2)	-7.94	2.38E-12
V-myc avian myelocytomatosis viral oncogene homolog(MYC)	-4.04	1.06E-08
Fibroblast growth factor receptor 3(FGFR3)	-3.94	8.54E-09
Thrombospondin 3(THBS3)	-3.22	3.34E-09
Fibronectin 1(FN1)	-2.98	9.41E-11
Camp responsive element binding protein 3 like 1(CREB3L1)	-2.65	8.79E-10
Tenascin C(TNC)	-2.39	1.07E-08
Platelet derived growth factor D(PDGFD)	-2.23	2.92E-07
Filamin C(FLNC)	-2.2	2.29E-07
Growth hormone receptor(GHR)	-2.16	9.01E-09
Laminin subunit gamma 2(LAMC2)	-2.15	1.69E-07
Collagen type IV alpha 6 chain(COL4A6)	-2.14	1.86E-08

Heparan sulfate proteoglycan 2(HSPG2)	-2.05	3.97E-06
KIT ligand(KITLG)	-1.98	1.87E-06
Protein phosphatase 2 regulatory subunit Bbeta(PPP2R2B)	-1.97	4.86E-07
Collagen type VI alpha 3 chain(COL6A3)	-1.91	0.0001
Ras protein specific guanine nucleotide releasing factor 1(RASGRF1)	-1.88	0.0002
Collagen type V alpha 2 chain(COL5A2)	-1.86	2.65E-06
SHC adaptor protein 3(SHC3)	-1.8	0.0004
Prolactin receptor(PRLR)	-1.8	1.20E-06
Cyclin dependent kinase 6(CDK6)	-1.68	3.57E-05
Collagen type IV alpha 5 chain(COL4A5)	-1.67	2.24E-06
Cyclin D1(CCND1)	-1.66	0.0013
Collagen type VI alpha 6 chain(COL6A6)	-1.65	0.0003
CD36 molecule(CD36)	-1.6	1.06E-05
Caveolin 1(CAV1)	-1.57	1.88E-07
Laminin subunit alpha 2(LAMA2)	-1.57	0.0001
Vascular endothelial growth factor B(VEGFB)	-1.55	1.49E-07
Cyclin D2(CCND2)	-1.53	4.73E-05
Laminin subunit alpha 5(LAMA5)	-1.52	0.0004
Laminin subunit gamma 1(LAMC1)	-1.52	9.53E-06

Protein phosphatase 2 regulatory subunit b"alpha(PPP2R3A)	-1.51	6.81E-05
Mechanistic target of rapamycin(MTOR)	1.51	8.90E-06
CD47 molecule(CD47)	1.53	1.01E-05
Vascular endothelial growth factor A(VEGFA)	1.56	4.93E-07
CRK proto-oncogene, adaptor protein(CRK)	1.57	0.0013
Ephrin A5(EFNA5)	1.57	7.62E-07
Syndecan 4(SDC4)	1.57	0.0001
Ribosomal protein S6 kinase B1(RPS6KB1)	1.58	0.0004
Integrin subunit alpha 2(ITGA2)	1.6	2.21E-07
BCL2 like 11(BCL2L11)	1.61	1.34E-05
CD44 molecule (Indian blood group)(CD44)	1.61	2.12E-05
Forkhead box O3(FOXO3)	1.61	2.17E-05
Phosphatase and tensin homolog(PTEN)	1.61	0.0005
Cyclin dependent kinase inhibitor 1B(CDKN1B)	1.7	0.0048
Interleukin 4 receptor(IL4R)	1.84	2.65E-07
Insulin receptor substrate 1(IRS1)	1.87	2.21E-06
Secreted phosphoprotein 1(SPP1)	2.41	7.68E-08
Interleukin 6 receptor(IL6R)	2.71	8.40E-08
